# Quality of life among cervical cancer patients undergoing radiotherapy

**DOI:** 10.11604/pamj.2020.35.125.18245

**Published:** 2020-04-17

**Authors:** Kofi Adesi Kyei, Frederick Yakanu, Andrew Donkor, Doris Kitson-Mills, Samuel Yaw Opoku, Joel Yarney, Samuel Nii Tagoe, Michael Kwabeng Addo, Kwabena Kwarko Anarfi, Eric Abakuri, Kofi Agyri

**Affiliations:** 1 Department of Radiography, School of Biomedical and Allied Health Sciences, College of Health Science, University of Ghana, Accra, Ghana; 2 National Centre for Radiotherapy and Nuclear Medicine, Accra, Ghana; 3 School of Health Sciences, Walden University, Minneapolis, USA; 4 Faculty of Health, University of Technology Sydney, Ultimo, Australia

**Keywords:** Cervical cancer, quality of life, assessment, FACT-G questionnaire

## Abstract

**Introduction:**

There has been an increasing rate of the incidence and mortality of cervical cancer in Ghana. Cancer and the treatment's side effects have adverse effects on the patients and this affects patient's well-being and lifestyle during and after radiotherapy. The study sought to assess the impact of demographic and clinical characteristics on Quality of Life (QoL) among cervical cancer patients undergoing radiotherapy in Ghana.

**Methods:**

A cross sectional quantitative study design was carried out on 120 cervical cancer patients who were conveniently sampled from the study site. The data was collected between the months of December, 2017 and February, 2018. QoL was measured using the FACT-G questionnaire. The mean scores of QoL were determined, whiles the chi-square test was used to determine the impact of socio-demographic and clinical characteristics on the QoL of the patients.

**Results:**

The mean age of the patients was 56.8 years. Majority of the patients reported stable QoL. The social well-being of the older patients was more affected than other patients. The unmarried, widows and patients who underwent surgery with radiotherapy were emotionally affected. Majority (56%) of the participants had stable QoL whiles 22% each had poor and good QoL. Significant association was found among 35-39 age group with physical well-being and overall QoL (p=0.017 and 0.029) respectively.

**Conclusion:**

There is a need to embrace a QoL assessment instrument in the study site so as to help the oncology team in the identification and addressing of specific indicators that affect the QoL of cervical cancer patients.

## Introduction

It is imperative to improve the quality of life of cervical cancer patients in low and middle income countries. Of the 279 thousand global cervical cancer deaths in 2015, 85% occurred in low and middle income countries (LMICs) [1]. Annually, cervical cancer accounts for 8.9 million disability-adjusted life years (DALYs), making it one of the leading causes of years lived with disability in LMICs [1]. Like other LMICs, the mortality burden of cervical cancer in Ghana is significantly increasing. In the absence of a formal cancer registry in 2012, it was estimated that 3052 new cases of cervical cancer were diagnosed in Ghana, with more than 50% deaths annually [2]. This morbidity and mortality burdens are expected to increase to 5,000 new cases of cervical cancer and 67.2 annual mortality rates. However, this growing burden can be prevented or reduced by investing the cost-effective and quality improvement strategies related to prevention, early detection, diagnosis, treatment (surgery, radiotherapy and essential cancer medicines), survivorship and palliative care [3]. Studies have been published concerning the health of Ghanaian cervical cancer patients and survivors [4-8] and these focused on the prevention, screening and population´s knowledge of the disease.

However, none of these studies has shown the effect of treatment on the QoL of cervical cancer patients. This introduces a gap in literature hence within the protocols in cancer treatment procedures especially cervical cancer. Apart from the gap in literature in Ghana, studies from other settings have indicated other contradicting outcomes with QoL [9,10]. A study done in Thai, showed that the emotional well-being of cervical cancer patients were influenced by age due to the mentality of life after death by the older ones [9]. The Thai cervical cancer patients experienced low anxiety about death; hence older women achieved high QoL. However, a similar study conducted by Miller *et al.* [10] in America reported contradicting outcome among the cervical cancer patients. In addition to the above studies, findings from a qualitative study conducted by Ashing-Giwa, Lim and Gonzalez [11] revealed significant association between the physical well-being and ethnicity. Hence, socio-demographic variables like race and cultural beliefs is seen to play a critical role in the setting protocols and treatment procedures for patients undergoing radiotherapy [12]. The study was aimed to assess impact of demographic and clinical characteristics on QoL of Ghanaian cervical cancer patients undergoing radiotherapy at the study site.

## Methods

A cross sectional quantitative study design was used in this study using clinically diagnosed cervical cancer patients undergoing radiotherapy at the study site. The study took place at the main Oncology Centre in Accra, Ghana between the months of December, 2017 and February, 2018. The centre was chosen for the study because it serves a total of 70% of all cancer cases seen in the country according to Kyei *et al.* [13]. One hundred and twenty cervical cancer patients were recruited for the study using a convenient sampling method [14]. The study included cervical cancer patients undergoing radiotherapy, with or without other treatment modalities such as chemotherapy, surgery and hormonal therapy. Patients who consented to participate in the study were selected. Non-Ghanaians undergoing cervical cancer treatment were excluded because of the difficulties in language barriers. Out of the 120 cervical cancer patients who took part in the study, none of them requested for withdrawal during the administration of questionnaire. FACT-G questionnaires were administered to the patients as it produced results which were reproducible for this type of study [15].

The questionnaires were made available to patients in English because, it is the only official language in Ghana. Quantitative data collected from the study was uploaded into a computerized database using Statistical Package for Social Sciences (SPSS) version 20.0. Descriptive statistics was used to analyze the socio-demographic characteristics such as age, level of education, marital status, clinical characteristics including the treatment modalities and stages of cancer as well as the QoL scores. Chi-square was used to determine the existence of association between socio-demographic and clinical characteristics with QoL. A p-value of 0.05 was used to define the chosen level of statistical significance. A Pearson´s chi-square with p-value less than 0.05 (p<0.05) signals significant difference between the variables and p-value equal or more than 0.05 (p≥0.05) indicates no significant difference between the variables. Confidentiality of all the information provided by the participants was ensured throughout collection and storage of data. Ethical approval was sought and obtained from the Ethics and Protocol Review Committee of a higher institution and the head of department of the study site before data collection. Consent was sought and obtained from all the 120 patients who took part in the study.

## Results

In all, 120 patients were recruited to participate in the study after consent and 100% response rate was achieved. Ages of participants ranged from 36 years to 76 years and majority of the participants, 24.2% (n=29) were within the age range of 55 years to 59 years. In Table 1, 42.5% (n=51) had no formal education, while 10% (n=12) attained higher education level. Almost 66% (n=79) were married while 4.2% (n=5) were single. As shown in Table 2, 44.2% (n=53) had stage III and only 2.5% (n=3) presented with stage I. Seventy percent (n=84) had combination of chemotherapy and radiotherapy where as 15.3% (n=19) had triple treatment modalities (surgery, chemotherapy and radiotherapy). The assessment of the QoL of the participants was determined using FACT-G questionnaire. For the purpose of this study, these initials were used: PWB- physical well-being, SWB- social well-being, EMB- emotional well-being, FWB- Functional well-being, and OQoL- grand quality of life.

**Table 1:** Demographic and clinical data (n=120)

Variables	Categories	Frequency (%)
Age	35-39	6 (5.0)
	40-44	15 (12.5)
	45-49	25 (20.8)
	50-54	21 (17.5)
	54-59	29 (24.2)
	60-64	9 (7.5)
	65-69	10 (8.3)
	70-74	3 (2.5)
	75-79	2 (1.7)
Education	Elementary	22 (18.3)
	Middle school	16 (13.3)
	Secondary	19 (15.9)
	Higher education	12 (10.0)
	No formal education	51 (42.5)
Marital Status	Single	5 (4.2)
	Married	79 (65.8)
	Divorced	26 (21.7)
	Widow	10 (8.3)

The majority of participants were between the ages of 54-59. The majority (42.5%) had no formal education. 65.8% were married during the time of treatment

**Table 2:** Clinical characteristics of patients n=120

Variables	Categories	Frequency (%)
Cancer Stage	Stage I	3 (2.5)
	Stage II	41 (34.2)
	Stage III	53 (44.2)
	Stage IV	23 (19.1)
Treatment Modalities	Radiotherapy only	14 (11.7)
	Radiotherapy & Chemotherapy only	84 (70.0)
	Radiotherapy & Surgery	3 (2.5)
	Radiotherapy, Chemotherapy & Surgery	19 (15.3)

The majority (63.3%) had stages three and beyond, with 70% of the patients undergoing a combination of chemotherapy and radiotherapy.

With the exception of social well-being in which 50-54 age group scored the highest mean score (19.0 ± 5.2), participants in the 35-37 age group scored highest mean scores in all the QoL items including OQoL mean score (Table 3). In the case of level of education, participants in the tertiary category scored the highest physical (19 ± 5.1), social (18.4 ± 5.3), emotional (17.2 ± 5.8), functional (21.6 ± 4.3) and overall QoL (76.6 ± 17.4) mean scores. Participants in the non-formal education category scored least in all the scores (Table 3). Married women scored the highest mean scores in all categories whiles the unmarried women scored least in the category of physical (9.5 ± 3.5) and emotional (5.0 ± 5.7) well-being and overall QoL (41.5 ± 7.8) mean scores (Table 3). In Table 4, treatment modalities were presented. Participants who went through combination of chemotherapy and radiotherapy scored the highest mean scores in physical (13.4 ± 5.9), functional, well-being (13.7 ± 5.3) and OQoL (55.5 ± 18.7). The highest mean scores were found among participants with stage II. Participants with stage IV had least mean scores in all categories.

**Table:** Mean and Pearson’s Chi-square P-value of participants’ quality of life

	PWB Mean (P-value)	SWB Mean (P-value)	EWB Mean (P-value)	FWB Mean (P-value)	OQo LMean (P-value)
**Age (n)**					
35-39 (6)	22.0 (0.017)	17.0 (0.947)	21.5 (0.063)	21.5 (0.198)	82.0 (0.029)
40-44 (15)	16.4 (0.166)	17.0 (0.763)	12.2 (0.493)	15.8 (0.088)	61.4 (0.636)
45-49 (25)	16.2 (0.336)	18.6 (0.490)	13.8 (0.890)	13.4 (0.920)	62.0 (0.636)
50-54 (21)	15.5 (0.097)	19.0 (0.101)	16.0 (0.112)	19 (0.003)	71.3 (0.539)
55-59 (29)	11.1 (0.714)	14.5 (0.443)	12.4 (0.778)	12.6 (0.611)	51.5 (0.539)
60-64 (9)	10.0 (0.331)	11.8 (0.332)	11.2 (0.224)	11.3 (0.167)	42.1 (0.228)
65-69 (10)	10.7 (0.826)	14.6 (0.787)	10.4 (0.583)	9.9 (0.318)	45.7 (0.323)
70-74 (3)	12.0 (0.845)	14.3 (0.629)	12.7 (0.621)	12.3 (0.751)	51.2 (0.651)
75-79 (2)	8.0 (0.176)	7.5 (0.003)	7.5 (0.991)	6.5 (0.560)	29.5 (0.029)
**Education (n)**					
Elementary (22)	13.4 (0.954)	16.4 (0.866)	14.2 (0.728)	13.1 (0.497)	57.2 (0.609)
Middle School (16)	14.7 (0.703)	14.7 (0.593)	12.7 (0.443)	14.1 (0.539)	56.3 (0.485)
Secondary (19)	13.6 (0.136)	16.4 (0.308)	11.4 (0.122)	13.1 (0.705)	54.5 (0.185)
Higher education (12)	19.4 (0.049)	18.4 (0.391)	17.2 (0.094)	21.6 (0.002)	76.6 (0.122)
No formal education (51)	10.0 (0.219)	13.2 (0.856)	11.3 (0.455)	11.1 (0.030)	45.7 (0.263)
**Marital Status (n)**					
Unmarried (5)	9.5 (0.997)	15.0 (0.424)	5.0 (0.037)	12.0 (0.961)	41.5 (0.968)
Married (79)	14.1 (0.623)	15.9 (0.515)	14.2 (0.269)	14.5 (0.431)	58.0 (0.508)
Divorced (26)	10.2 (0.730)	12.6 (0.614)	10.4 (0.203)	10.0 (0.361)	45.1 (0.313)
Widow (10)	11.0 (0.505)	14.8 (0.803)	10.0 (0.014)	12.8 (0.960)	48.3 (0.412)

Significant association was found among 35-39 age group with physical well-being and overall QoL (p= 0.017 and 0.029 respectively.

**Table 4:** Quality of life with cancer stages and treatment modalities (n=120)

	PWB Mean (P-value)	SWB Mean (P-value)	EWB Mean (P-value)	FWB Mean (P-value)	OQoL Mean (P-value)
**Stages (n)**					
Stage I (3)	16.0 (0.770)	17.0 (0.816)	18.0 (0.925)	14.0 (0.897)	65.0 (0.858)
Stage II (41)	16.9 (0.440)	17.6 (0.318)	15.7 (0.197)	17.3 (0.021)	67.5 (0.400)
Stage III (53)	11.6 (0.512)	14.4 (0.121)	12.4 (0.888)	11.6 (0.518)	49.5 (0.310)
Stage IV (23)	8.1 (0.249)	12.0 (0.050)	7.5 (0.204)	10.0 (0.761)	38.7 (0.554)
**Treatment Modalities (n)**					
Radiotherapy only (14)	12.9 (0.569)	14.1 (0.233)	13.3 (0.201)	13.4 (0.188)	53.5 (23.6)
Radiotherapy & Chemotherapy (84)	13.4 (0.800)	15.5 (0.176)	13.2 (0.409)	13.7 (0.602)	55.5 (0.485)
Surgery + Radiotherapy (3)	12.0 (1.000)	18.0 (0.816)	5.0 (0.000)	12.0 (0.897)	47.0 (0.994)
Radiotherapy, Chemotherapy & Surgery (19)	9.3 (0.601)	12.8 (0.575)	9.8 (0.851)	11.0 (0.754)	44.8 (0.220)

A strong evidence of association was indicated between participants who underwent surgery and radiotherapy and emotional well-being (p= 0.000).

## Discussion

It was evident from the study that incidence of cervical cancer increased with age and then dropped at a peak of 55 to 59 year group. In terms of knowledge about the cancer disease and its preventive measures in Africa, the younger women are better placed than the older women due to the current ongoing education and public awareness in the country. This was consistent with other studies conducted in other parts of Ghana and also in Kenya, where authors commented that, cervical cancer was prevalent among age group of premenopausal to menopausal women [16,17]. This study further affirms that cervical cancer was not dominant among the younger participants unlike breast cancers [13].

### Impact of demographic and clinical characteristics on QoL

**Physical well-being:** according to the findings of the study, the physical well-being of cervical cancer patients was affected by their age and the level of education (Table 3). It was identified that the majority had no formal education. This was significant in identifying whether or not their interpretations of their condition and the effect of treatment was spot-on. Again, the findings might be due to the attention and worry of the younger and the educated patients on the side effects of the treatment. In effect, one´s level of education and their age could influence their overall outlook and this was in line with a study by Ashing-Giwa *et al.* [11] where their findings suggested a significant association between level of education and physical well-being among breast and cervical cancer patients.

**Social well-being:** this study reported no significant association between the treatment modalities and the social well-being of the patients (Table 3). Hence, the kind of treatment did not suggestively affect the socio-familial life of the patients. However, a study by Frumovitz *et al.* [18] found that sexual dysfunction significantly affected the social life of the participants in their study. Furthermore, another study showed that patients with physical changes such as fatigue, hair loss, darkening of skin and weight loss as a result of side effect of their treatment had their social lives affected [19]. Therefore, the consequences of the physical changes could be stigmatization, isolation from social milieu and loneliness [20].

**Emotional well-being:** emotionally, married women scored higher in terms of mean score than unmarried women (Table 3). The presence of spouse or partners played a role in the well-being of the patients emotionally. This study reported significant association between marital status and emotion of the patients. Specifically, emotional impact was found among patients who were single and widowed, thus these findings could be due to the absence of partners to occupy periods of loneliness and also assist in re-assurance during the treatment. Despite high emotional well-being score among patients with early staged cancer, stage of the cancer did not have any significant effect on emotional well-being of the patients in this setting. To support this finding, Pasek *et al.* [21] also reported no significant association between emotional well-being and cancer stage. In consistent with the current study, Baze *et al.* [22] reported that, due to the severity of the disease, poor emotional scores were recorded among advanced cancer staged patients. Meanwhile, Azmawati *et al.* [23] reported high emotional well-being scores among patients with advanced stage of cancer. The current study revealed significant association between treatment modalities and emotional well-being. Additionally, patients who underwent combination of surgery and radiotherapy were likely to face more emotional impact compared to other groups (Table 4). Thus, Perrin *et al.* [24] stipulated feelings of fear, hopelessness, anger, shock and self-blame as the outcomes of physical changes as a result of the treatment. However, Frumovit *et al.* [18] reported better emotional well-being among patients treated with surgery only compared to those treated with radiotherapy only. Krikeli *et al.* [25] revealed that emotional well-being had no significant impact on the patients no matter the kind of treatment the patient underwent.

**Functional well-being:** patients within the age range of 50 to 54 years had significant impact on their functional well-being (Table 3). In concord with the current study, Greimel *et al.* [26] reported no significant difference among all their age groups. The study reported significant association between patients with cancer stage II and functional well-being (Table 4). This by implication means only patients with cancer stage II are likely to be affected functionally. Patients who underwent chemotherapy and radiotherapy were functionally stronger and had no statistically significant association between the kind of treatment and functional well-being. Hence, the functional well-being had no effect on the kind of treatment the patients underwent. In contrast, Greimel *et al.* [26] reported contradicting outcome on functional well-being.

**Overall quality of life:** early staged cancer patients reported with higher overall QoL scores than those with late presentation of the disease. The finding of the current study was in accordance with that of other studies [23-25]. There was no statistically significant relationship between the forms of treatment received by patients and their overall QoL. Thence, patients were unlikely to be significantly affected by the kind of treatment they underwent. The current study was consistent with another similar study [27]. Yet, other contrary findings were present in another similar study [18]. Also, no significant association was found between cancer stage and overall QoL of the patients. Hence, irrespective of the stage of the disease, patients were not functionally retarded. However, contradicting findings were present in other studies reported by Ogoncho *et al.* [17]. The difference in outcomes could be due to the differences in the presentation of the disease. Usually, the developing countries presents with late cancer stage compared to the early stage presentation of disease in the develop countries [13].

## Conclusion

The findings of this study showed that, majority of cervical cancer patients receiving treatment at the study site had stable QoL. While younger patients were likely to be affected physically during the course of the treatment, functional challenges were possible among menopausal patients and socio-familial challenges among older patients. During the course of the treatment, stage II cervical cancer patients were likely to be affected functionally. Hence, some socio-demographic and clinical characteristics were likely to affect the QoL of the patients. There is a need to embrace a QoL assessment instrument at the study site so as to help the oncology team in the identification and addressing of specific indicators that affect the QoL of cervical cancer patients.

### What is known about this topic

- A study conducted among Ghanaian cervical cancer patients reported 64% presenting with advanced stage of the disease;
- Reports indicate that older cervical patients who are unmarried have positive correlation with QoL;
- Higher levels of education correlate with higher QoL and the differences are clear in the social and functional domain.

### What this study adds

- Cervical cancer is not dominant among the younger age grouped below 35 years;
- Education and age affected the physical well-being of cervical cancer patients;
- Early cervical cancer patients reported with higher overall QoL scores than those with late presentation of the disease.
